# New Benzenoid Derivatives and Other Constituents from *Lawsonia inermis* with Inhibitory Activity against NO Production

**DOI:** 10.3390/molecules22060936

**Published:** 2017-06-05

**Authors:** Chang-Syun Yang, Jih-Jung Chen, Hui-Chi Huang, Guan-Jhong Huang, Sheng-Yang Wang, Ping-Jyun Sung, Ming-Jen Cheng, Ming-Der Wu, Yueh-Hsiung Kuo

**Affiliations:** 1 Department of Chinese Pharmaceutical Sciences and Chinese Medicine Resources, China Medical University, Taichung 404, Taiwan; tim.tim0619@msa.hinet.net (C.-S.Y.); hchuang@mail.cmu.edu.tw (H.-C.H.); gjhuang@mail.cmu.edu.tw (G.-J.H.); 2 Faculty of Pharmacy, School of Pharmaceutical Sciences, National Yang-Ming University, Taipei 112, Taiwan; chenjj@ym.edu.tw; 3 Department of Medical Research, China Medical University Hospital, China Medical University, Taichung 404, Taiwan; 4 Department of Forestry, National Chung-Hsing University, Taichung 402, Taiwan; taiwanfir@dragon.nchu.edu.tw; 5 Agricultural Biotechnology Research Center, Academia Sinica, Taipei 115, Taiwan; 6 National Museum of Marine Biology and Aquarium, Pingtung 944, Taiwan; pjsung@nmmba.gov.tw; 7 Food Industry Research and Development Institute, Hsinchu 300, Taiwan; low463@gmail.com (M.-J.C.); wmd@firdi.org.tw (M.-D.W.); 8 Department of Biotechnology, Asia University, Taichung 413, Taiwan

**Keywords:** *Lawsonia inermis*, lawsoinermone, inermidioic acid, inermic acid, inhibitory activities against NO production

## Abstract

Three new benzenoid derivatives, lawsoinermone (**1**), inermidioic acid (**2**), and inermic acid (**3**) have been isolated from the aerial part of *Lawsonia inermis*, together with 11 known compounds (**4**–**14**). The structures of three new compounds were determined through spectroscopic and MS analyses. Compounds **1**, **4**–**6**, **13** and **14** were evaluated for inhibition of nitric oxide production in LPS-stimulated product of nitrite in RAW 264.7 cells with IC_50_ values of 6.12, 16.43, 18.98, 9.30, 9.30 and 14.90 μg/mL, respectively.

## 1. Introduction

*Lawsonia inermis* Linn (Lythraceae) is a small tree or tall shrub, native to northern Africa, western and southern Asia, and northern Australasia [1]. *Lawsonia inermis* is a folk herbal medicine used for skin diseases and as a wound drug in Taiwan [2]. Isocoumarins [3], flavonoids [3,4], quinoids [4], triterpenoids [4], naphthalene derivatives [4], coumarins [4], and their derivatives are widely distributed in plants of the family Lythraceae. Many of these compound derivatives exhibit anti-inflammatory [3,5], antimycotic, antifungal, antibacterial, and antiparasitic activities [6]. In our studies on the anti-inflammatory constituents of Formosan plants, many species have been screened for in vitro inhibitory activity on macrophage pro-inflammatory responses, and *L. inermis* has been found to be an active species. The current phytochemical investigation of the aerial part of this plant has led to the isolation of three new compounds—lawsoinermone (**1**), inermidioic acid (**2**), and inermic acid (**3**)—along with 11 known compounds. The structural elucidation of **1**–**3** and the anti-inflammatory activity of the isolates are described herein.

## 2. Results and Discussion

### 2.1. Isolation and Structural Elucidation

**Figure 1:**
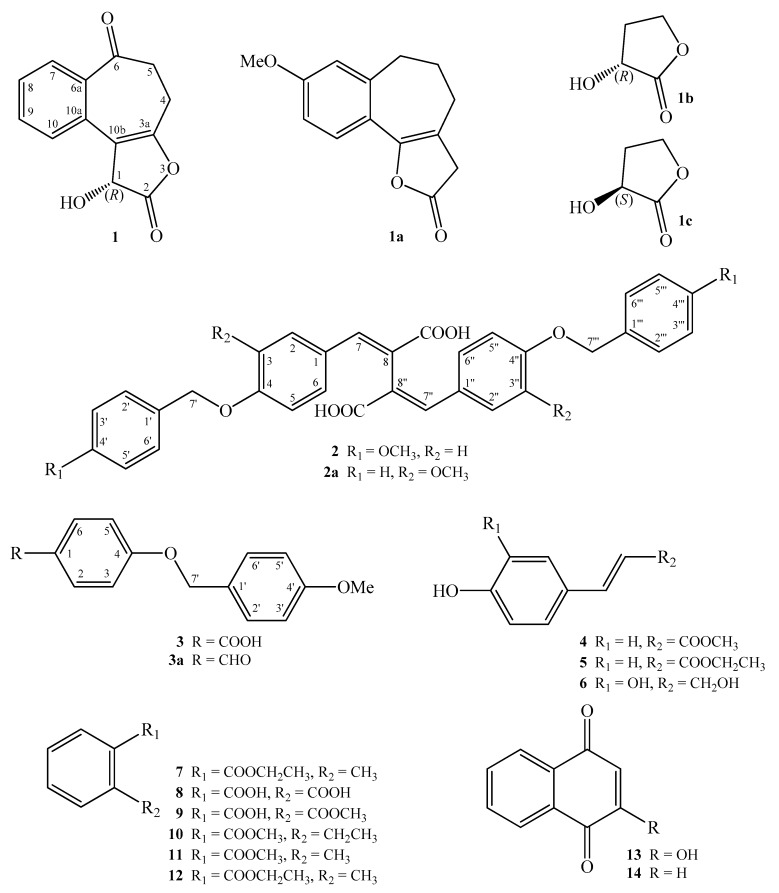
The chemical structures of compounds **1**–**14**.

The MeOH extract of the aerial part of *Lawsonia inermis* was concentrated to give a brown-green residue, which was suspended in water and partitioned with EtOAc and H_2_O, successively. The combined EtOAc soluble fraction was purified by repeated silica gel column chromatography and normal phase semipreparative high-performance liquid chromatography (HPLC) to obtain 3 new benzenoid derivatives—lawsoinermone (**1**), inermidioic acid (**2**), and inermic acid (**3**)—and 11 known compounds **4**–**14** (Figure 1).

**Figure 2:**
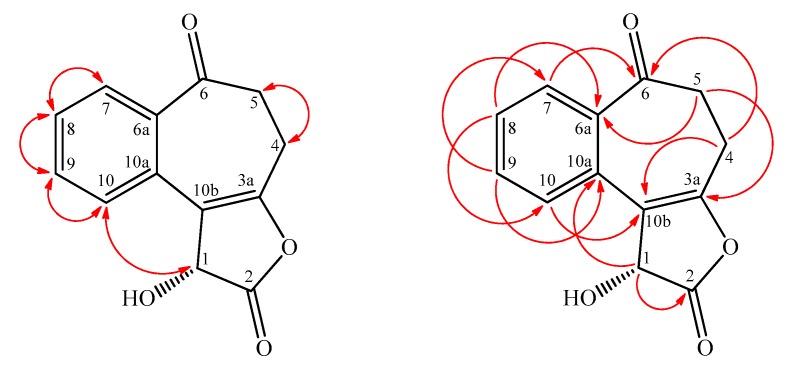
Key NOESY (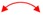) and HMBC (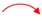) correlations of **1**.

Lawsoinermone (**1**) was isolated as light yellow oil with molecular formula C_13_H_10_O_4_ as determined by HR-ESI-MS, showing an [M − H]^−^ ion at *m*/*z* 229.0498 (calcd. for C_13_H_9_O_4_, 229.0495). The IR absorption bands implied the presence of an OH (3442 cm^−1^), a γ-butyrolactone carbonyl group (1772 cm^−1^), and a conjugated carbonyl group (1682 cm^−1^). The ^1^H-NMR spectrum of **1** showed the presence of four mutually coupling aromatic protons [δ 7.46 (1H, br t, *J* = 7.8 Hz, H-8), 7.60 (1H, br t, *J* = 7.8 Hz, H-9), 7.85 (1H, br d, *J* = 7.8 Hz, H-7), 8.55 (1H, br d, *J* = 7.8 Hz, H-10)], four mutually coupling methylene protons [δ 2.79 (1H, m, H-4α), 2.95 (1H, m, H-4β), and 3.07 (2H, m, H-5)], and an oxymethine proton [δ 6.07 (1H, s, H-1)], and a hydroxyl group [δ 3.88 (1H, br s, D_2_O exchangeable, OH-1)]. The ^1^H-NMR spectrum of **1** was similar to 3,4,5,6-tetrahydro-8-methoxy-2*H*-benzo[6,7] cyclohepta[*b*]furan-2-one (**1a**) [7], except that H-8, α-hydroxy-γ-butyrolactone moiety at C-3a and C-10b, and a ketone at C-6 of **1** replaced OMe-8, γ-butyrolactone moiety at C-3a and C-10b, and H-6 of **1a** [7]. This was supported by (1) NOESY correlations observed between H-1 (δ_H_ 6.07)/H-10 (δ_H_ 8.55), H-8 (δ_H_ 7.46)/H-7 (δ_H_ 7.85), and H-8 (δ_H_ 7.46)/H-9 (δ_H_ 7.60), and (2) HMBC correlations observed between H-1 (δ_H_ 6.07)/C-2 (δ_C_ 169.1), H-1 (δ_H_ 6.07)/C-10a (δ_C_ 127.2), H-4 (δ_H_ 2.79, 2.95)/C-6 (δ_C_ 201.0), H-8 (δ_H_ 7.46)/C-6a (δ_C_ 138.9), and H-8 (δ_H_ 7.46)/C-10 (δ_C_ 128.9). Furthermore, the absolute configuration of **1** was proposed to be *R* by comparing specific rotation data ${\left[\alpha \right]}_{D}^{20}$ +59.6° (*c* 1.20, CHCl_3_) of **1** with those reported for (*R*)-3-hydroxydihydrofuran-2(3*H*)-one (**1b**) (${\left[\alpha \right]}_{D}^{25}$ +61.5° (*c* 1.15, CHCl_3_)) [8] and (*S*)-3-hydroxydihydrofuran-2(3*H*)-one (**1c**) (${\left[\alpha \right]}_{D}^{24}$ −64.8° (*c* 1.82, CHCl_3_)) [9]. The full assignment of ^1^H- and ^13^C-NMR resonances was confirmed by ^1^H-^1^H COSY, NOESY (Figure 2), DEPT, HSQC, and HMBC (Figure 2) techniques. According to the evidence above, the structure of **1** was elucidated as (*R*)-1-hydroxy-4,5-dihydro-1H-benzo[3,4]cyclohepta[1,2-*b*]furan-2,6-dione, named lawsoinermone.

**Figure 3:**
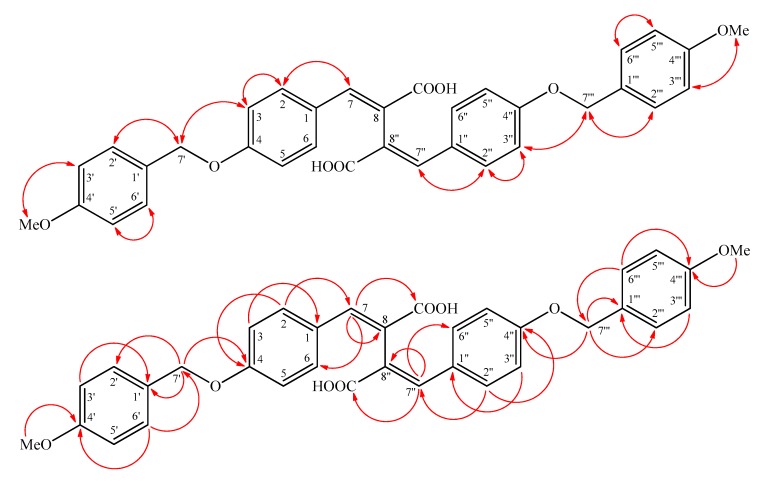
Key NOESY (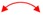) and HMBC (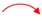) correlations of **2**.

Inermidioic acid (**2**) was obtained as yellow powder. Its molecular formula, C_34_H_30_O_8_, was determined on the basis of the positive HR-ESI-MS at *m*/*z* 567.6049 [M + H]^+^ (calcd. for C_34_H_31_O_8_, 567.6051) and supported by the ^1^H, ^13^C, and DEPT NMR data. The presence of conjugated carboxyl group was revealed by the bands at 3300~2500 and 1679 cm^−1^ in the IR spectrum, and was confirmed by the resonance at δ 167.8 in the ^13^C-NMR spectrum. The ^1^H-NMR spectrum of **2** displayed the presence of a 4-methoxybenzyloxy moiety [δ 3.74 (3H, s, OMe-4′), 4.98 (2H, s, H-7′), 6.92 (2H, d, *J* = 8.4 Hz, H-3′ and H-5′), 7.33 (2H, d, *J* = 7.8 Hz, H-2′ and H-6′)], four aromatic protons on para-disubstituted benzene ring [δ 6.95 (2H, d, *J* = 8.6 Hz, H-3 and H-5) and 7.49 (2H, d, *J* = 8.6 Hz, H-2 and H-6)], and a conjugated olefinic proton [δ 7.71 (1H, s, H-7)]. Based on the HR-ESI-MS, ^1^H-, and ^13^C-NMR data, the number of resonances observed was half that expected, suggesting that **2** had a symmetrical structure. The ^1^H-NMR data of **2** were similar to 2,3-bis(4-benzyloxy-3-methoxybenzylidene)succinic acid [8], except that H-3/H-3′′ (δ 6.95) and OMe-4′/OMe-4′′′ groups (δ 3.74) of **2** replaced OMe-3/OMe-3′′ and H-4′/H-4′′′ of 2,3-bis(4-benzyloxy-3-methoxybenzylidene)succinic acid (**2a**) [10]. This was supported by the HMBC correlations between OMe-4′ (δ_H_ 3.74)/C-4′ (δ_C_ 159.1) and OMe-4′′′ (δ_H_ 3.74)/C-4′′′ (δ_C_ 159.1) of **2**, and by the NOESY correlations between OMe-4′ (δ_H_ 3.74)/H-3′ (δ_H_ 6.92), OMe-4′ (δ_H_ 3.74)/H-5′ (δ_H_ 6.92), H-3 (δ_H_ 6.95)/H-2 (δ_H_ 7.49), H-3 (δ_H_ 6.95)/H-7′ (δ_H_ 4.98), H-3′′ (δ_H_ 6.95)/H-2′′ (δ_H_ 7.49), and H-3′′ (δ_H_ 6.95)/H-7′′′ (δ_H_ 4.98) of **2**. Compound **2** showed the similar UV absorption [265 nm] and the similar chemical shift [δ 7.71] of H-7 and H-7′′ when compared to the analogous (2*E*,3*E*)-2,3-bis(4-(benzyloxy)benzylidene)succinic acid [11], and the (2*E*,3*E*)-configuration of **2** was thus established. On the basis of the above data, the structure of **2** was elucidated as (2*E*,3*E*)-2,3-bis(4-(4′-methoxybenzyloxy)benzylidene)succinic acid and named inermidioic acid. This was further confirmed by the ^1^H-^1^H COSY, NOESY (Figure 3), DEPT, HSQC, and HMBC (Figure 3) techniques.

**Figure 4:**
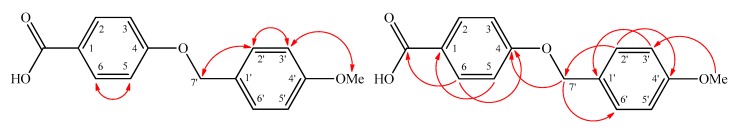
Key NOESY (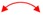) and HMBC (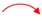) correlations of **3**.

Inermic acid (**3**) was isolated as amorphous powder with molecular formula C_15_H_14_O_4_ as determined by HR-EI-MS, showing an [M]^+^ ion at *m*/*z* 258.0901 (calcd. for C_15_H_14_O_4_, 258.0893). The presence of carboxyl group in **3** was revealed by the bands at 3300~2500 and 1682 cm^−1^ in the IR spectrum. The ^1^H-NMR spectrum of **3** showed the presence of a 4-methoxybenzyloxy moiety [δ 3.83 (3H, s, OMe-4′), 5.06 (2H, s, H-7′), 6.92 (2H, d, *J* = 7.8 Hz, H-3′ and H-5′), 7.36 (2H, d, *J* = 7.8 Hz, H-2′ and H-6′)], four para-substituted aromatic protons [δ 7.01 (2H, d, *J* = 8.4 Hz, H-3 and H-5) and 8.05 (2H, d, *J* = 8.4 Hz, H-2 and H-6)], and a carboxyl group [δ 10.68 (1H, br s, D_2_O exchangeable, COOH-1)]. The ^1^H-NMR spectrum of **3** was similar to that of 4-(4-methoxybenzyloxy)-benzaldehyde (**3a**) [12], except that the 1-carboxyl group of **3** replaced 1-formyl group of **3a** [10]. This was supported by the HMBC correlations observed between H-2/H-6 (δ_H_ 8.05) and COOH (δ_C_ 169.8). The full assignment of ^1^H- and ^13^C-NMR resonances was confirmed by ^1^H-^1^H COSY, NOESY (Figure 4), DEPT, HSQC, and HMBC (Figure 4) techniques. According to the evidence above, the structure of **3** was elucidated as 4-(4-methoxybenzyloxy) benzoic acid. This is the first report of the occurrence of **3** in a natural source, although it has been synthesized by Mosley [13].

### 2.2. Structure Identification of the Known Isolates

The known isolates were readily identified by a comparison of physical and spectroscopic data (UV, IR, ^1^H-NMR, ^1^^3^C-NMR, and MS) with corresponding authentic samples or literature values, and this included nine benzenoids, (*E*)-methyl 3-(4-hydroxyphenyl)acrylate (**4**) [14], (*E*)-ethyl 3-(4-hydroxyphenyl)acrylate (**5**) [15], caffeoyl alcohol (**6**) [16], ethyl 2-methylbenzoate (**7**) [17], benzene-1,2-dicarboxylic acid (**8**) [18], monomethyl *ortho*-phthalate (**9**) [19], methyl 2-ethylbenzoate (**10**) [20], methyl 2-methylbenzoate (**11**) [18], and ethyl 2-methylbenzoate (**12**) [21], and two naphthoquinones—2-hydroxy-1,4-naphthoquinone (**13**) [21] and 1,4-naphthoquinone (**14**) [22].

### 2.3. Inhibitory Activity against Nitric Oxide Production

Nitric oxide (NO) is derived from the oxidation of l-arginine by NO synthase (NOS) and is a mediator in the inflammatory response involved in host defense [23]. In inflammation and carcinogenesis conditions, there is an increased production of NO by inducible NO synthase (iNOS) [24]. In this study, the inhibitory activity toward NO production of 3 new (**1**–**3**) and 11 known compounds (**4**–**14**) was evaluated by measurement of nitrite/nitrate in LPS-stimulated RAW 264.7 cells. To search for the appropriate concentrations for the above assay, these 14 compounds were first tested for their cytotoxic activity against the RAW 264.7 cells, and no significant cytotoxic activities were observed under all tested concentrations. From the results of our anti-inflammatory tests, the following conclusions could be drawn: (a) The high cell viability (>92%) indicated that the inhibitory activities of compounds **1**, **4**, **5**, **6**, **13** and **14** on LPS-induced NO production did not resulted from their cytotoxicities; (b) Compounds **1**, **6** and **13** exhibited inhibitory effects on lipopolysaccharides (LPS)-induced nitric oxide production in RAW 264.7 cells with IC_50_ values of 6.12 ± 2.84, 9.30 ± 4.26, and 9.30 ± 4.68 μg/mL, respectively (Table 1); (c) lawsoinermone (**1**) is the most effective among the isolated compounds, with IC_50_ = 6.12 ± 2.84 μg/mL, against LPS-induced NO generation.

## 3. Experimental Section

### 3.1. General

Optical rotations were measured using a Jasco P-1020 polarimeter (Jasco, Kyoto, Japan) in CHCl_3_. Ultraviolet (UV) spectra were obtained with a Shimadzu Pharmaspec-1700 UV-Visible spectrophotometer (Shimadzu, Kyoto, Japan). Infrared (IR) spectra (neat or KBr) were recorded on a Shimadzu IR prestige-21 Fourier transform infrared spectrophotometer (Shimadzu, Kyoto, Japan). Nuclear magnetic resonance (NMR) spectra—including correlation spectroscopy (COSY), nuclear Overhauser effect spectrometry (NOESY), heteronuclear multiple-bond correlation (HMBC), and heteronuclear single-quantum coherence (HSQC) experiments—were recorded on a Bruker DRX-500 FT-NMR (Bruker, Bremen, Germany) operating at 500 MHz (^1^H) and 125 MHz (^13^C), respectively, with chemical shifts given in ppm (**δ**) using tetramethylsilane (TMS) as an internal standard. Mass spectrometric (HR-EI-MS) data were generated at the Mass Spectrometry Laboratory of the Chung Hsing University (Taichung, Taiwan). Column chromatography was performed using LiChroCART Si gel (5 μM; Merck, Darmstadt, Germany), and TLC analysis was carried out using aluminum pre-coated Si plates (Merck, Darmstadt, Germany) and the spots were visualized using a UV lamp at λ = 254 nm.

### 3.2. Chemicals

The solvents used to open column isolation (Sephadex LH 20 and silica gel column) in the study, such as *n*-hexane, chloroform, ethyl acetate, acetone, and methanol were as ACS grade. The HPLC grade *n*-hexane, ethyl acetate, and acetone for HPLC isolation and the deuterated solvent for NMR measurement (CDCl_3_, acetone-*d*_6_, or CD_3_OD) were purchased from the branch of Merck in Taipei, Taiwan. LPS (endotoxin from *Escherichia coli*, serotype 0127:B8), Carr (type IV), indomethacin, MTT (3-[4,5-dimethylthiazol-2-yl]-2,5-diphenyltetrazolium bromide) and other chemicals were purchased from Sigma Chemical Co. (St. Louis, MO, USA).

### 3.3. Plant Material

*Lawsonia inermis* was collected from Neipu Township, Pingtung, Taiwan, in February 2009 and identified by I.-S. Chen (Emeritus Professor, School of Pharmacy, College of Pharmacy, Kaohsiung Medical University, Kaohsiung, Taiwan). A voucher specimen (CMU-LIY-090711) was deposited at the School of Chinese Pharmaceutical Sciences and Chinese Medicine Resources.

### 3.4. Extraction and Isolation

The dried aerial part (5.0 kg) of *Lawsonia inermis* was extracted three times with MeOH (50 L each) for seven days. The extract was concentrated under reduced pressure at 35 °C, and the residue (440 g) was partitioned between EtOAc and H_2_O (1:1) to provide the EtOAc-soluble fraction (fraction A; 132.5 g). Fraction A (132.5 g) was purified by column chromatography (CC) (6.0 kg of SiO_2_, 70–230 mesh; *n*-hexane/EtOAc/methanol gradient) to afford 14 fractions: A1–A14.

Fraction A3 (42.40 g) was re-separated by silica gel column chromatography (*n*-hexane:ethyl acetate = 8:1) and semi-preparative normal phase HPLC (*n*-hexane:acetone = 10:1) to afford pure compounds **1** (62.8 mg), **2** (157.3 mg), **3** (12.5 mg), **4** (16.4 mg), and **5** (5.3 mg). Fraction A5 (36.7 g) was re-separated by silica gel column chromatography (*n*-hexane:ethyl acetate = 6:1) and semi-preparative normal phase HPLC (*n*-hexane:acetone = 8:1) to afford pure compounds **6** (11.6 mg), **7** (32.4 mg), and **8** (23.4 mg). Fraction A8 (22.4 g) was re-separated by Sephadex LH 20 column chromatography (chloroform:methanol = 3:7), silica gel column chromatography (*n*-hexane:acetone = 8:3) and then semi-preparative HPLC (chloroform:acetone = 6:1) to afford pure compounds **9** (10.2 mg), **10** (17.5 mg), **11** (15.0 mg), **12** (23.4 mg), **13** (33.4 mg), and **14** (24.5 mg).

Lawsoinermone (**1**): light yellow oil; ${\left[\alpha \right]}_{D}^{20}$ +59.6° (*c* 1.20, CHCl_3_); UV (MeOH): λ_max_ (log ε) 290 (4.34), 265 (4.24), 211 (4.34); IR (KBr) υ_max_: 3442, 1772, 1682, 1607, 1502 cm^−1^; ^1^H-NMR (CDCl_3_, 500 MHz): δ 2.79 (1H, m, H-4α), 2.95 (1H, m, H-4β), 3.07 (2H, m, H-5), 3.88 (1H. br s, OH-1), 6.07 (1H. s, H-1), 7.46 (1H, br t, *J* = 7.8 Hz, H-8), 7.60 (1H, br t, *J* = 7.8 Hz, H-9), 7.85 (1H, br d, *J* = 7.8 Hz, H-7), 8.55 (1H, br d, *J* = 7.8 Hz, H-10); ^13^C-NMR (CDCl_3_, 125 MHz): δ 22.4 (C-4), 41.2 (C-5), 95.9 (C-1), 126.8 (C-10b), 127.2 (C-10a), 128.9 (C-10), 129.5 (C-8), 129.8 (C-7), 132.5 (C-9), 138.9 (C-6a), 160.3 (C-3a), 169.1 (C-2), 201.0 (C-6); ESI-MS *m*/*z* 229 [M − H]^−^; HR-ESI-MS *m*/*z* 229.0498 [M − H]**^−^** (calcd. for C_13_H_9_O_4_, 229.0495).

Inermidioic acid (**2**): yellow powder; UV (MeOH): λ_max_ (log ε) 291 (4.52), 265 (4.59); IR (KBr) υ_max_: 3300~2500, 1679, 1607, 1508 cm^−1^; ^1^H-NMR (CDCl_3_, 400 MHz): δ 3.74 (6H, s, OMe-4′ and OMe-4′′′), 4.98 (4H, s, H-7′ and H-7′′′), 6.92 (4H, d, *J* = 8.4 Hz, H-3′, H-5′, H-3′′′, and H-5′′′), 6.95 (4H, d, *J* = 8.6 Hz, H-3, H-5, H-3′′, and H-5′′), 7.33 (4H, d, *J* = 8.4 Hz, H-2′, H-6′, H-2′′′, and H-6′′′), 7.49 (4H, d, *J* = 8.6 Hz, H-2, H-6, H-2′′, and H-6′′), 7.71 (2H, s, H-7 and H-7′); ^13^C-NMR (CDCl_3_, 100 MHz): δ 55.1 (OMe-4′ and OMe-4′′′), 69.1 (C-7′ and C-7′′′), 113.8 (C-3′, C-5′, C-3′′′, and C-5′′′), 115.0 (C-3, C-5, C-3′′, and C-5′′), 125.8 (C-8 and C-8′′), 127.2 (C-1 and C-1′′), 128.5 (C-1′ and C-1′′′), 129.6 (C-2′, C-6′, C-2′′′, and C-6′′′), 131.4 (C-2, C-6, C-2′′, and C-6′′), 140.1 (C-7 and C-7′′), 159.1 (C-4′ and C-4′′′), 159.5 (C-4 and C-4′′), 167.8 (COOH); ESI-MS *m*/*z* 567 [M + H]^+^; HR-ESI-MS *m*/*z* 567.6049 [M + H]^+^ (calcd. for C_34_H_31_O_8_, 567.6051).

Inermic acid (**3**): amorphous powder; UV (MeOH): λ_max_ (log ε) 321 (4.26), 294 (4.33), 242 (4.31), 220 (4.43); IR (KBr) υ_max_: 3300~2500, 1682, 1628, 1578, 1530 cm^−1^; ^1^H-NMR (CDCl_3_, 500 MHz): δ 3.83 (3H, s, OMe-4′), 5.06 (2H, s, H-7′), 6.92 (2H, d, *J* = 7.8 Hz, H-3′ and H-5′), 7.01 (2H, d, *J* = 8.4 Hz, H-3 and H-5), 7.36 (2H, d, *J* = 7.8 Hz, H-2′ and H-6′), 8.05 (2H, d, *J* = 8.4 Hz, H-2 and H-6), 10.68 (1H, br s, COOH); ^13^C-NMR (CDCl_3_, 125 MHz): δ 55.3 (OMe-4′), 70.0 (C-7′), 114.1 (C-3′ and C-5′), 114.6 (C-3 and C-5), 121.5 (C-1), 128.1 (C-1′), 129.3 (C-2′ and C-6′), 132.3 (C-2 and C-6), 159.7 (C-4′), 163.2 (C-4), 169.8 (COOH); EI-MS *m*/*z* 258 [M]^+^; HR-EI-MS *m*/*z* 258.0901 [M]^+^ (calcd. for C_15_H_14_O_4_, 258.0893).

### 3.5. Cell Culture

A murine macrophage cell line RAW264.7 (BCRC No. 60001) was purchased from the Bioresources Collection and Research Center (BCRC, Hsinchu, Taiwan) of the Food Industry Research and Development Institute (Hsinchu, Taiwan). Cells were cultured in plastic dishes containing Dulbecco’s Modified Eagle Medium (DMEM, Sigma, St. Louis, MO, USA) supplemented with 10% fetal bovine serum (FBS, Sigma) in a CO_2_ incubator (5% CO_2_ in air) at 37 °C and subcultured every three days at a dilution of 1:5 using 0.05% trypsin-0.02% EDTA in Ca^2+^-, Mg^2+^-free phosphate-buffered saline (DPBS).

### 3.6. Cell Viability

Cells (2 × 10^5^) were cultured in 96-well plate containing DMEM supplemented with 10% FBS for one day to become nearly confluent. Then cells were cultured with compounds **1**–**14** in the presence of 100 ng/mL LPS (lipopolysaccharide) for 24 h. After that, the cells were washed twice with DPBS and incubated with 100 μL of 0.5 mg/mL MTT for 2 h at 37 °C testing for cell viability. The medium was then discarded and 100 μL dimethyl sulfoxide (DMSO) was added. After 30-min incubation, absorbance at 570 nm was read using a microplate reader (Molecular Devices, Sunnyvale, CA, USA).

### 3.7. Measurement of Nitric Oxide/Nitrite

NO production was indirectly assessed by measuring the nitrite levels in the cultured media and serum determined by a colorimetric method based on the Griess reaction. The cells were incubated with different concentration of samples in the presence of LPS (100 ng/mL) at 37 °C for 24 h. Then, cells were dispensed into 96-well plates, and 100 μL of each supernatant was mixed with the same volume of Griess reagent (1% sulfanilamide, 0.1% naphthylethylenediamine dihydrochloride and 5% phosphoric acid) and incubated at room temperature for 10 min, the absorbance was measured at 540 nm with a Micro-Reader (Molecular Devices, SpectraMax^®^ M2e, Sunnyvale, CA, USA). By using sodium nitrite to generate a standard curve, the concentration of nitrite was measured from absorbance at 540 nm.

### 3.8. Statistical Analysis

The data is expressed as means ± standard errors (SE). The IC_50_ values were calculated from the dose curves using a non-linear regression algorithm (SigmaPlot 8.0; SPSS Inc., Chicago, IL, USA, 2002). Statistical evaluation was carried out by one-way analysis of variance (ANOVA followed by Scheffe’smultiple range tests).

## Figures and Tables

**Table 1:** Inhibitory effect of compounds **1**–**14** on overproduction of nitric oxide in LPS-stimulated RAW 264.7 cells.

Compounds	IC_50_ (μg/mL) ^a^
**1**	6.12 ± 2.84
**2**	>20
**3**	>20
**4**	16.43 ± 2.68
**5**	18.98 ± 3.48
**6**	9.30 ± 4.26
**7**	>20
**8**	>20
**9**	>20
**10**	>20
**11**	>20
**12**	>20
**13**	9.30 ± 4.68
**14**	14.90 ± 3.86
Indomethacin ^b^	59.48 ± 1.22

^a^ The IC_50_ values were calculated from the slope of the dose-response curves (SigmaPlot). Values are expressed as average ± SEM (*n* = 3); ^b^ Indomethacin was used as a positive control.

## Supplementary Materials

Supplementary materials are available online, Figures S1–S7: MS, 1D, and 2D-NMR spectra for Lawsoinermone (**1**), Figures S8–S14: MS, 1D, and 2D-NMR spectra for Inermidioic acid (**2**), Figures S15–S21: MS, 1D, and 2D-NMR spectra for Inermic acid (**3**).
