# Supplementary material for: New Benzenoid Derivatives and Other Constituents from Lawsonia inermis with Inhibitory Activity against NO Production
Source: Molecules. 2017 Jun 5;22(6):936. doi: 10.3390/molecules22060936 (PMC6152715; doi:10.3390/molecules22060936)
Supplement: Supplementary file 1 [file molecules-22-00936-s001.pdf]

Supplementary Materials

New Benzenoid Derivatives and other Constituents from *Lawsonia inermis* with Inhibitory Activity against NO Production

Chang-Syun Yang [1,†], Jih-Jung Chen [2,3,†], Hui-Chi Huang [1], Guan-Jhong Huang [1,†], Sheng-Yang Wang [4,5], Ping-Jyun Sung [6], Ming-Jen Cheng [7], Ming-Der Wu [7], Yueh-Hsiung Kuo [1,8,*]

1 Department of Chinese Pharmaceutical Sciences and Chinese Medicine Resources, China Medical University, Taichung 404, Taiwan; tim.tim0619@msa.hinet.net (C.-S.Y.); hchuang@mail.cmu.edu.tw (H.-C.H.); gjhuang@mail.cmu.edu.tw (G.-J.H.); kuoyh@mail.cmu.edu.tw (Y.-H. K.)
2 Faculty of Pharmacy, School of Pharmaceutical Sciences, National Yang-Ming University, Taipei 112, Taiwan; chenjj@ym.edu.tw (J.-J. C.)
3 Department of Medical Research, China Medical University Hospital, China Medical University, Taichung 404, Taiwan
4 Department of Forestry, National Chung-Hsing University, Taichung 402, Taiwan; taiwanfir@dragon.nchu.edu.tw
5 Agricultural Biotechnology Research Center, Academia Sinica, Taipei 115, Taiwan
6 National Museum of Marine Biology and Aquarium, Pingtung 944, Taiwan; pjsung@nmmba.gov.tw
7 Food Industry Research and Development Institute, Hsinchu, 300, Taiwan; low463@gmail.com (M.-J.C); wmd@firdi.org.tw (M.-D.W)
8 Department of Biotechnology, Asia University, Taichung 413, Taiwan
* Correspondence: kuoyh@mail.cmu.edu.tw; Tel.: +886-4-2205-3366 (ext. 5701); Fax: +886-4-2207-1693
† These authors contributed equally to this work.

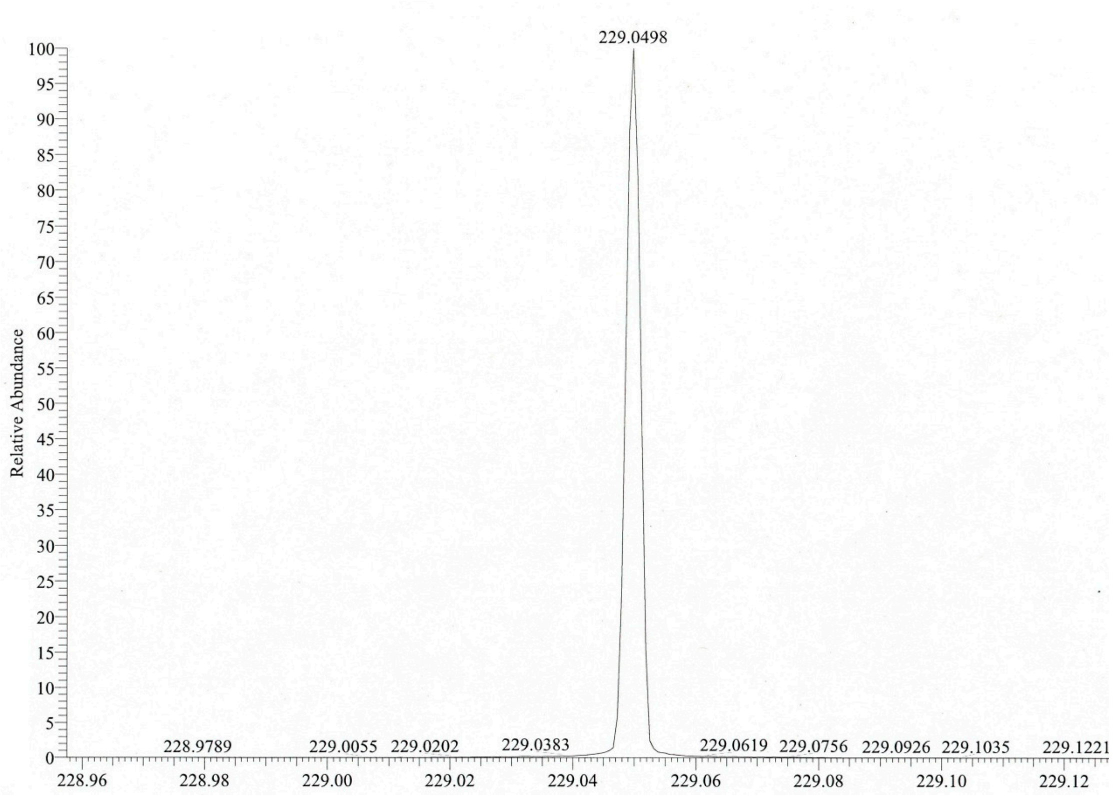

Fig. S1. HR-ESI-MS spectrum of **1**.

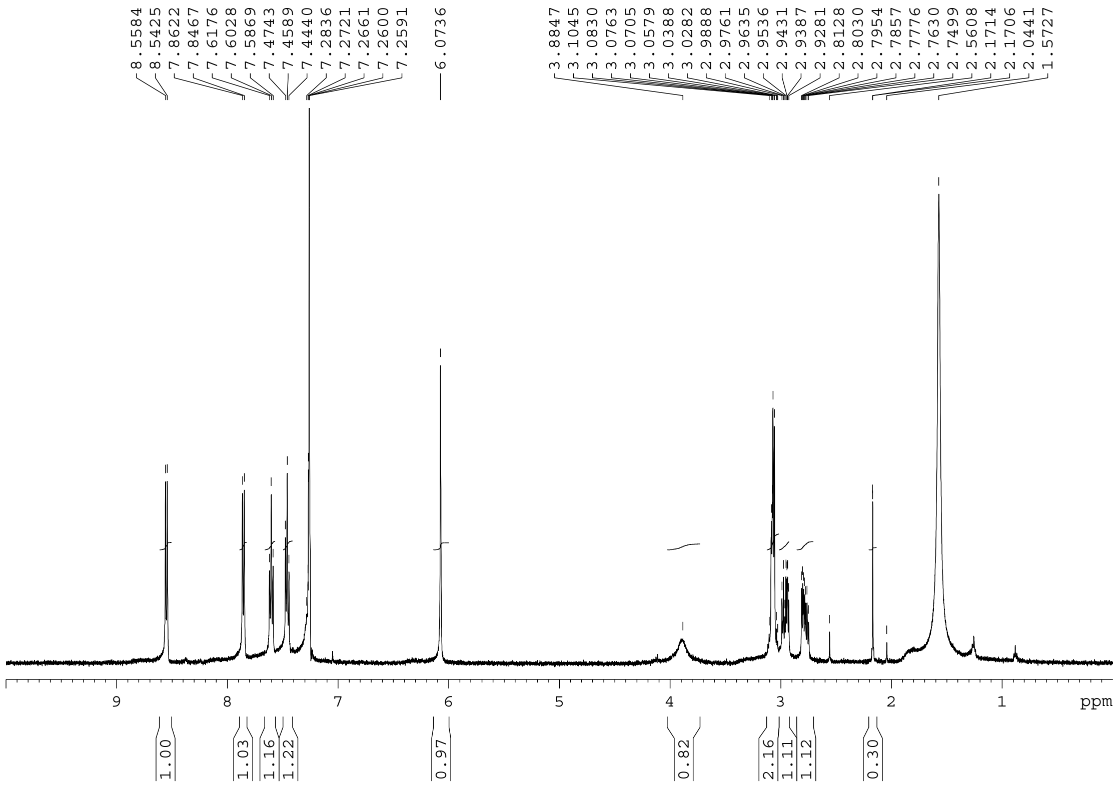

Fig. S2. ^{1}H-NMR spectrum of **1** ($CDCl_3$, 500 MHz).

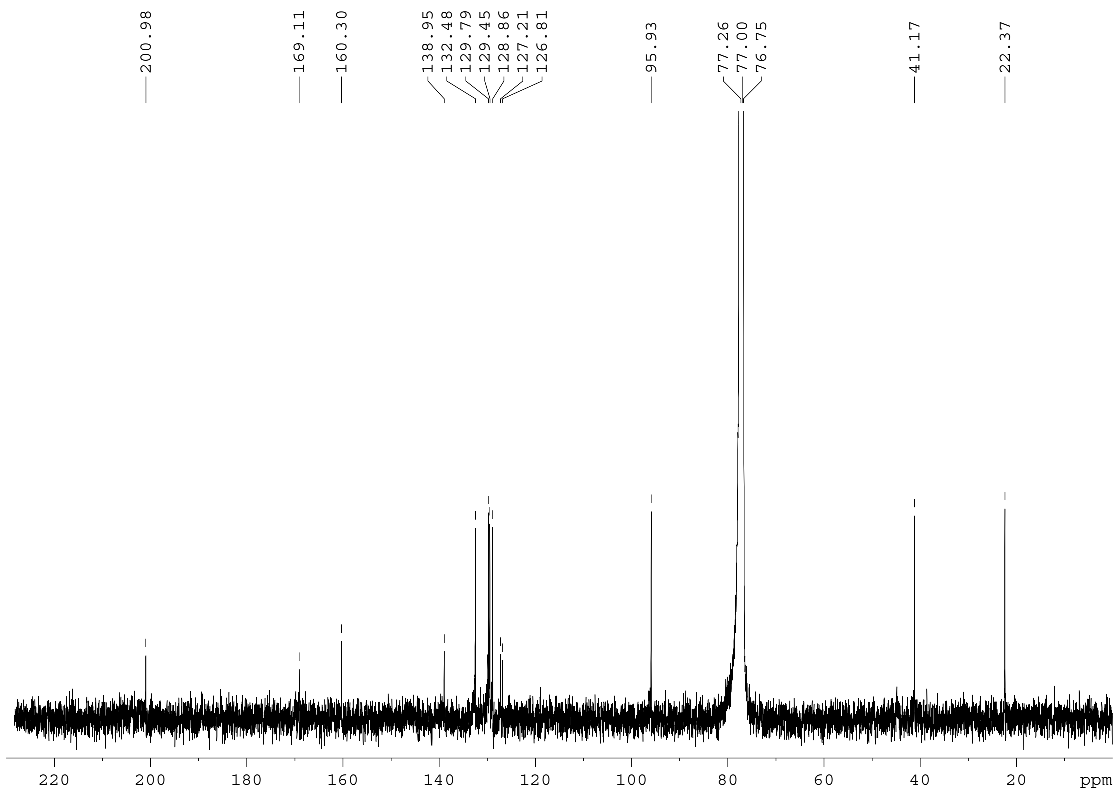

Fig. S3. ^{13}C-NMR spectrum of **1** ($CDCl_3$, 125 MHz).

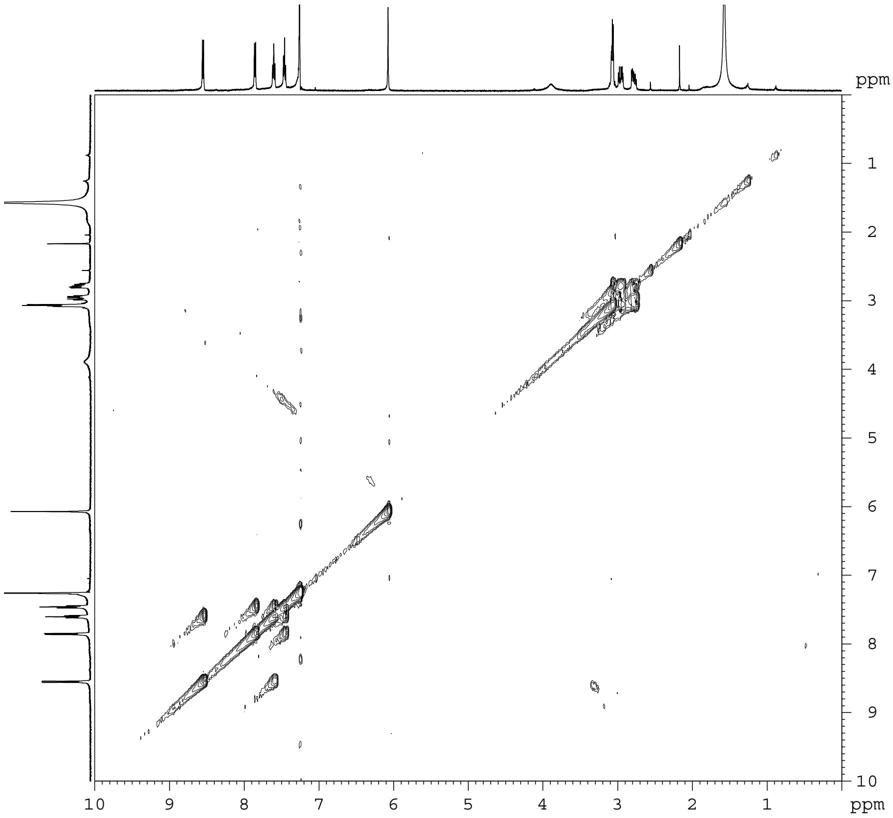

Fig. S4. ^{1}H–^{1}H COSY spectrum of **1**.

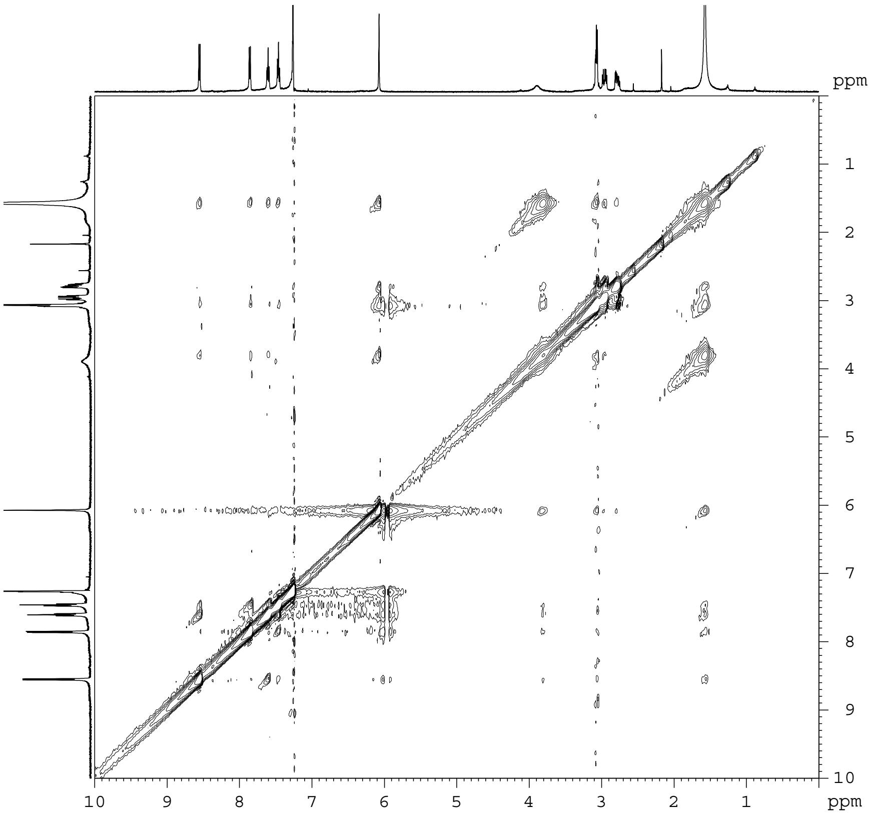

Fig. S5. NOESY spectrum of **1.**

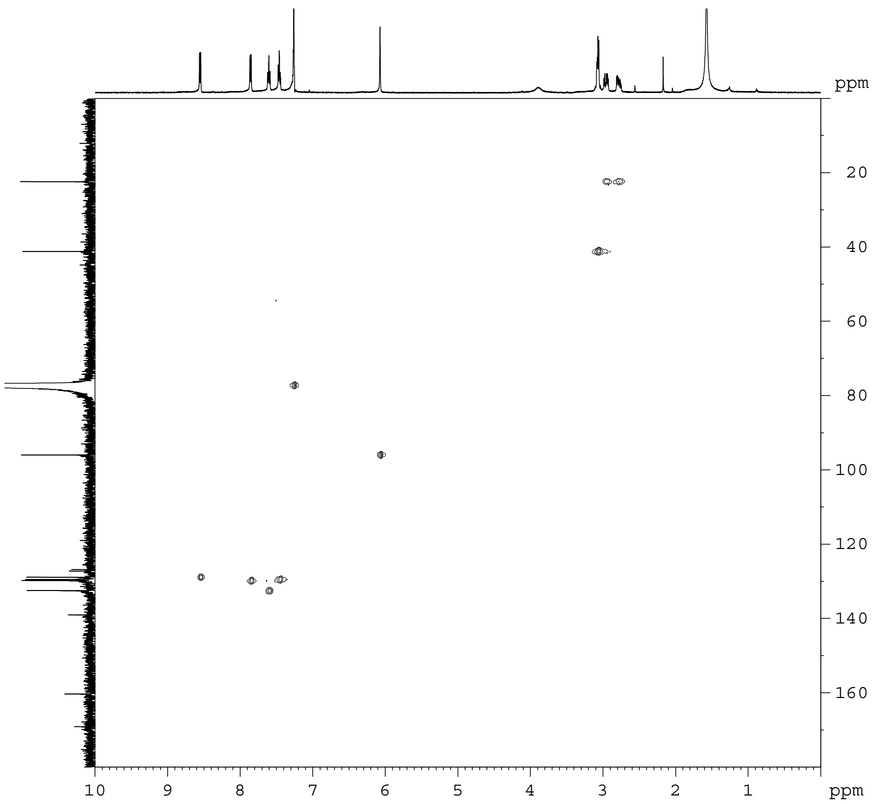

Fig. S6. HSQC spectrum of **1**.

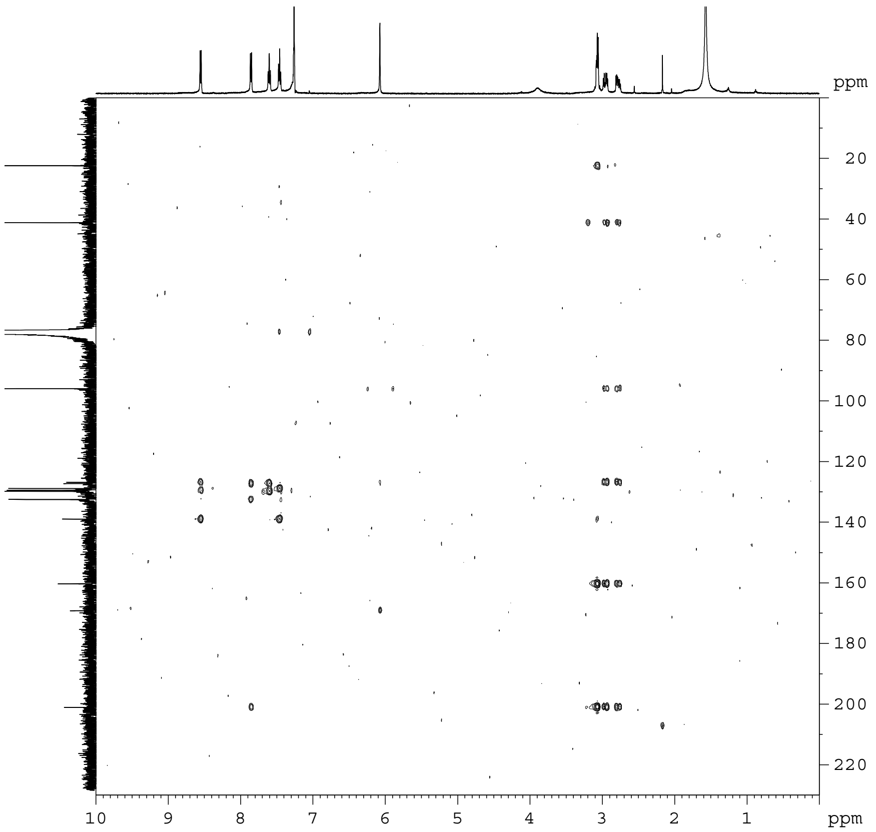

Fig. S7. HMBC spectrum of **1**.

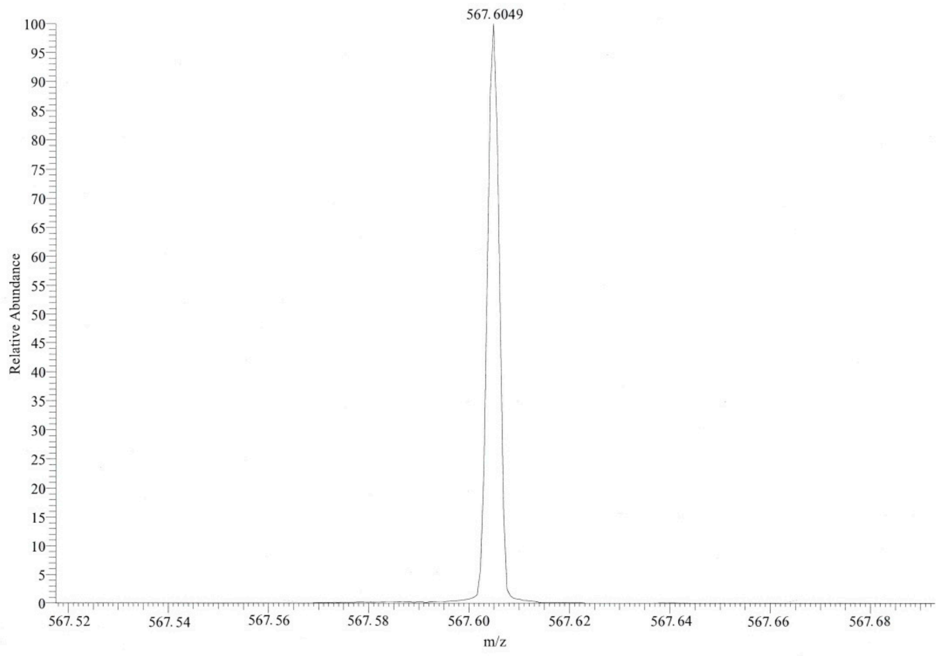

Fig. S8. HR-ESI-MS spectrum of **2**.

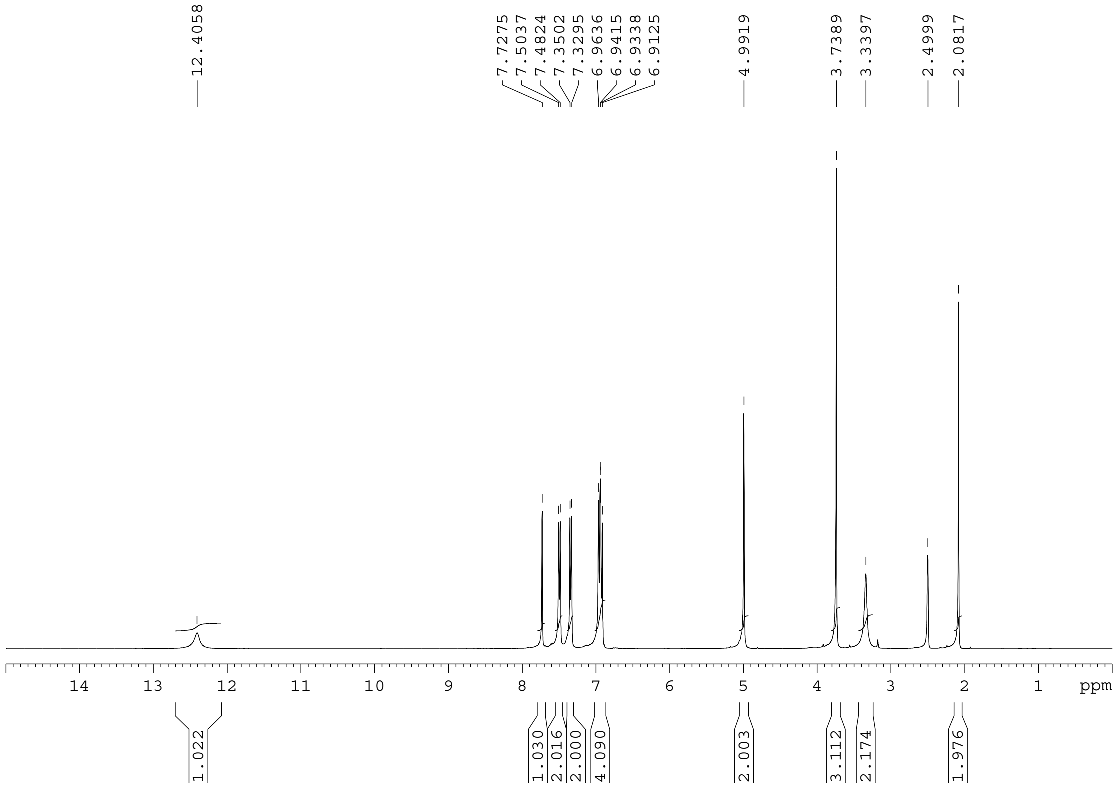

Fig. S9. ^{1}H-NMR spectrum of **2** ($CDCl_3$, 400 MHz).

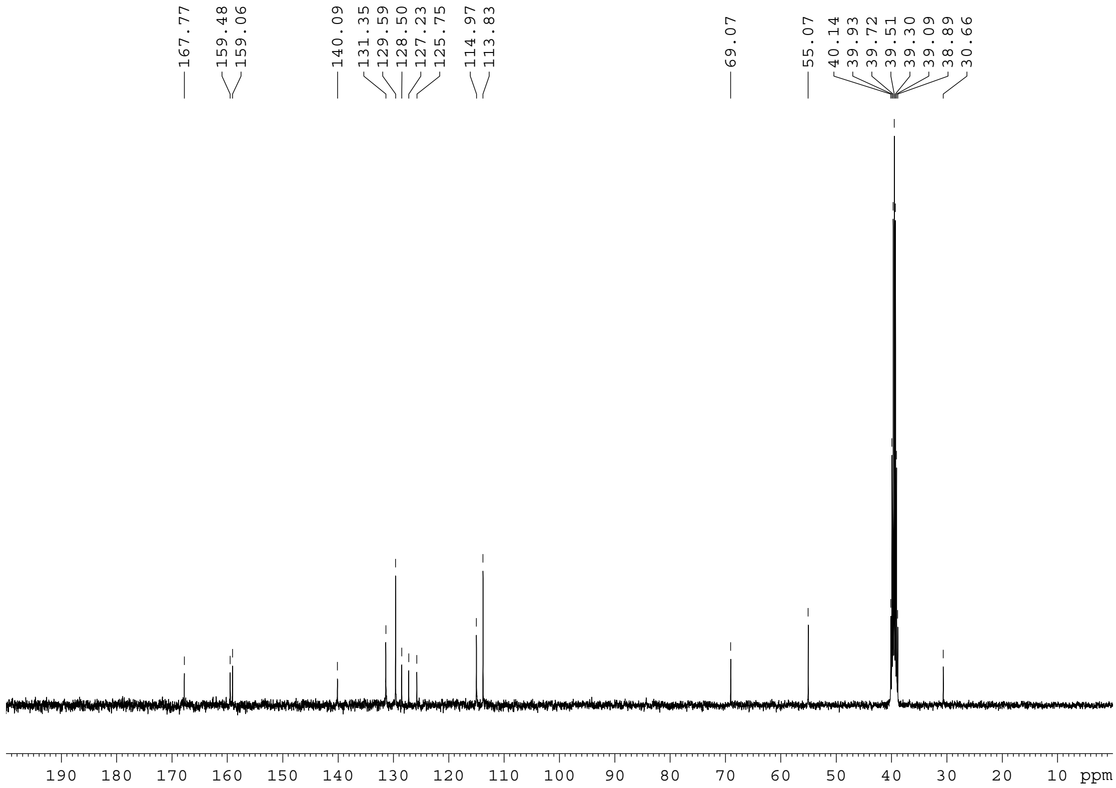

Fig. S10. ^{13}C-NMR spectrum of **2** ($CDCl_3$, 100 MHz).

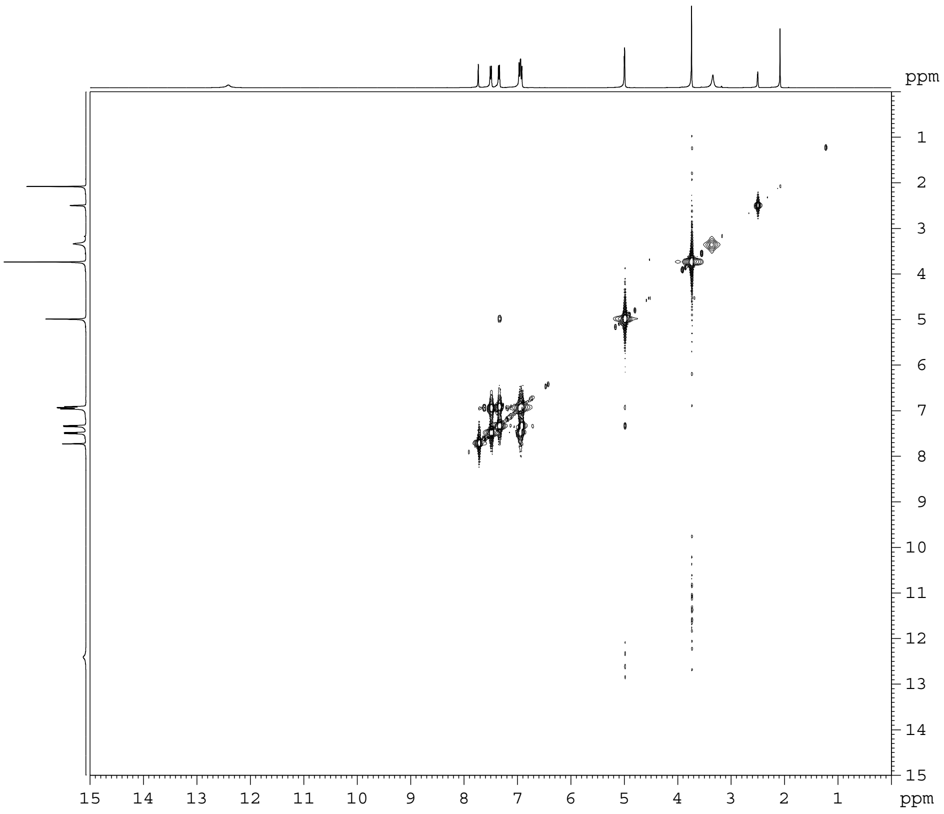

Fig. S11. ^{1}H–^{1}H COSY spectrum of **2**.

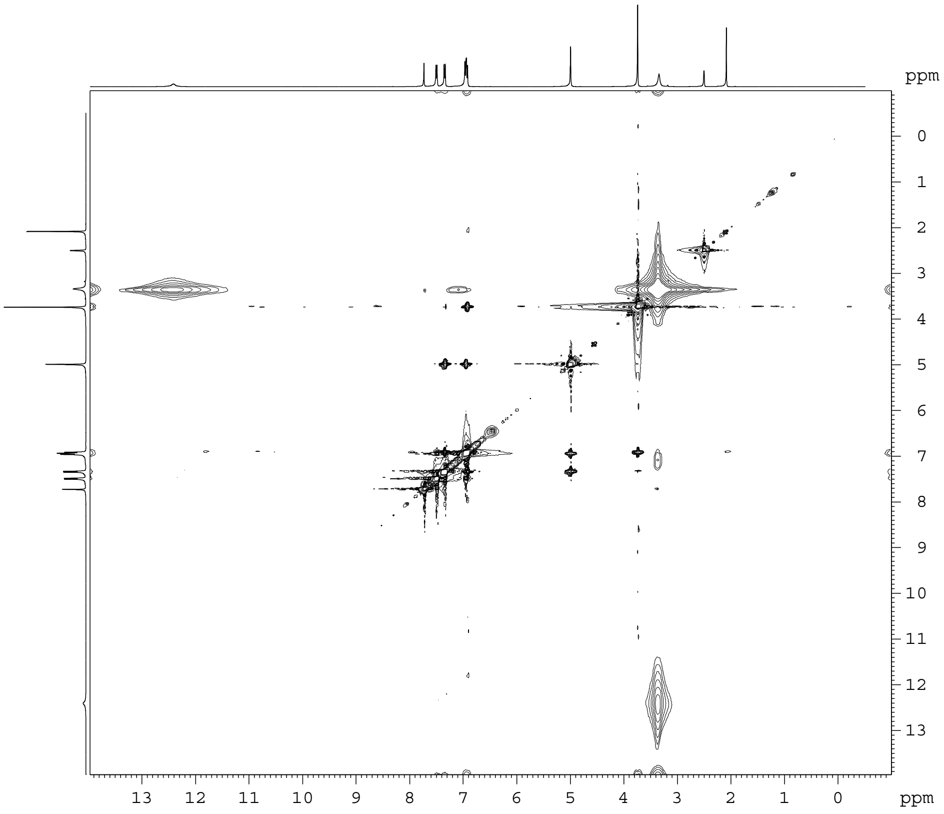

Fig. S12. NOESY spectrum of **2**.

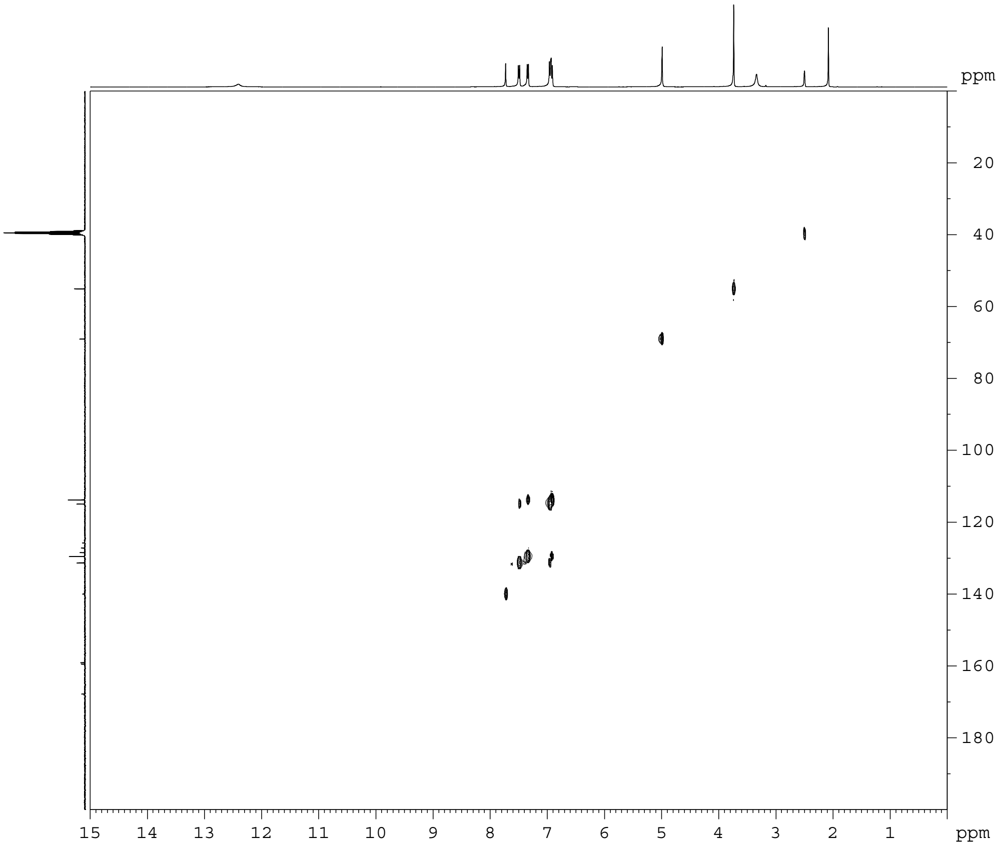

Fig. S13. HSQC spectrum of **2**.

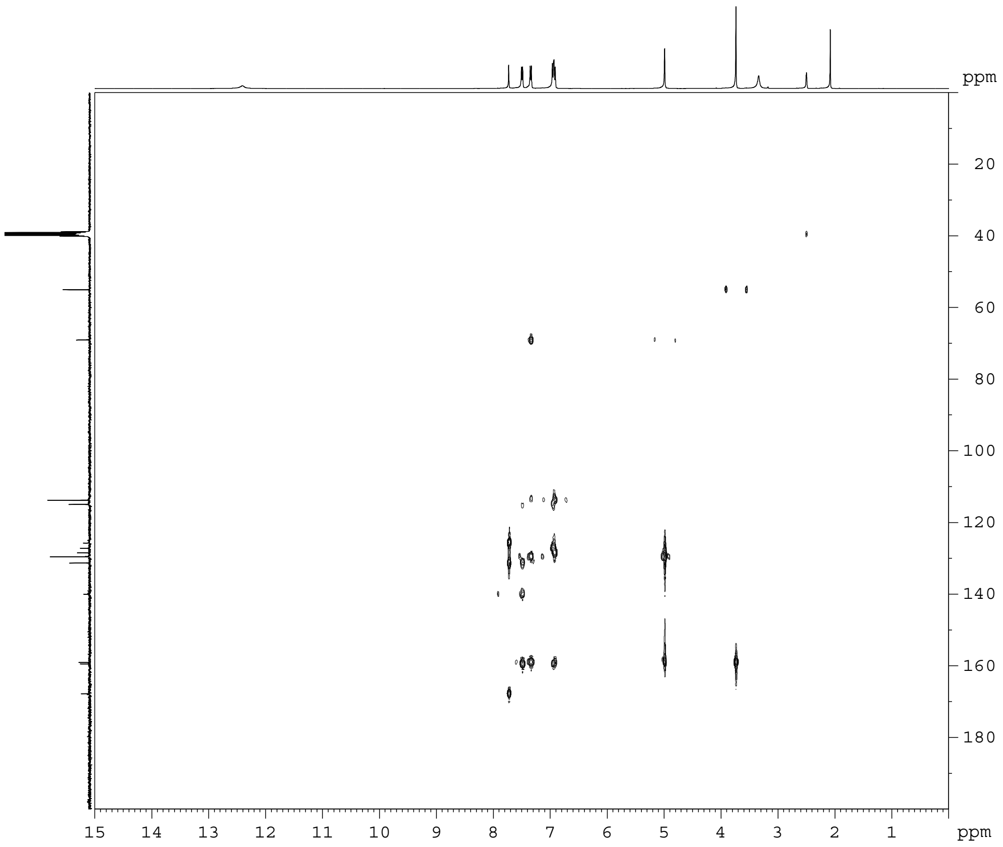

Fig. S14. HMBC spectrum of **2**.

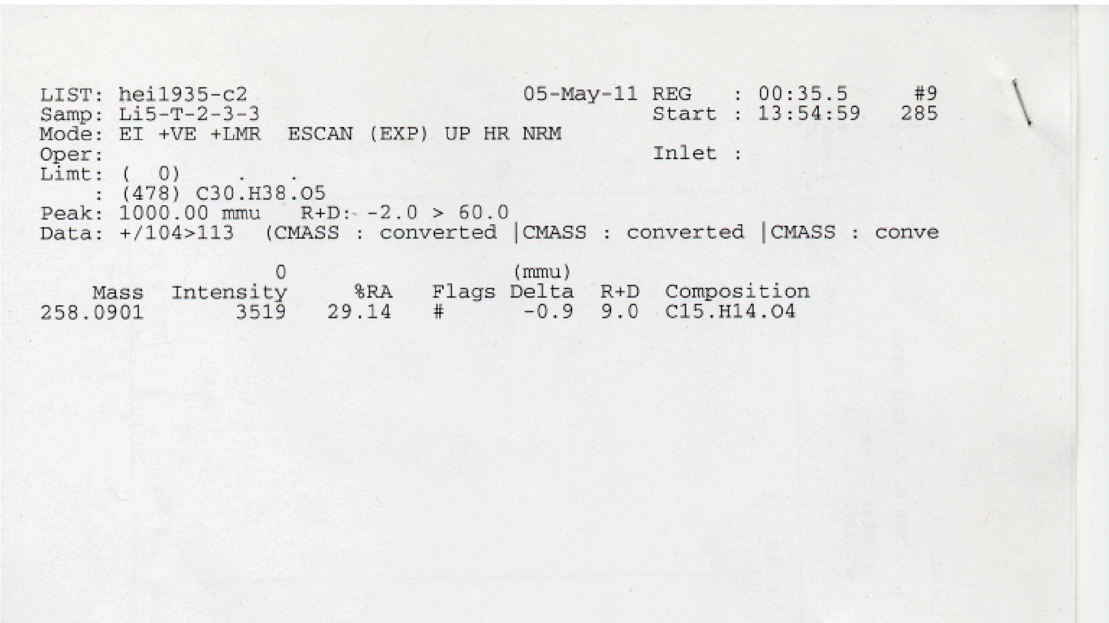

```
LIST: hei1935-c2                     05-May-11 REG    : 00:35.5     #9
Samp: Li5-T-2-3-3                              Start  : 13:54:59   285
Mode: EI +VE +LMR  ESCAN (EXP) UP HR NRM
Oper:                                          Inlet  :
Limt: (  0)      .   .
    : (478) C30.H38.O5
Peak: 1000.00 mmu   R+D: -2.0 > 60.0
Data: +/104>113  (CMASS : converted |CMASS : converted |CMASS : conve

                   0                 (mmu)
    Mass  Intensity     %RA   Flags Delta  R+D  Composition
258.0901       3519   29.14    #     -0.9  9.0  C15.H14.O4
```

Fig. S15. HR-EI-MS spectrum of **3**.

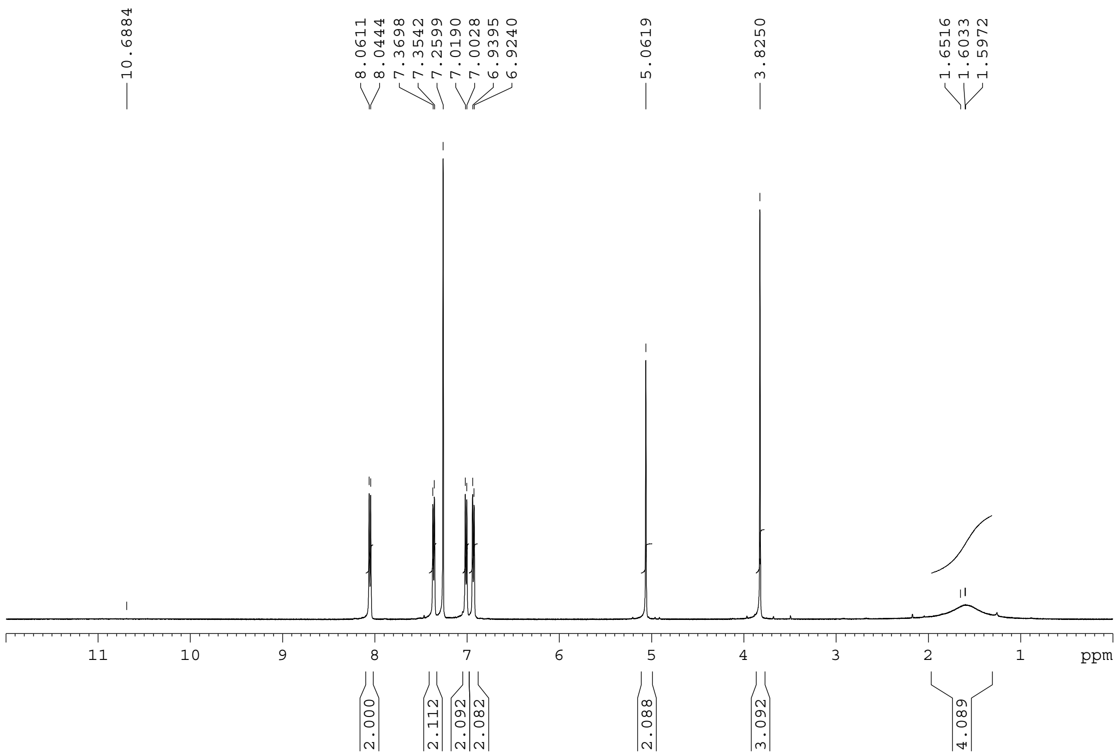

Fig. S16. ^{1}H-NMR spectrum of **3** ($CDCl_3$, 500 MHz).

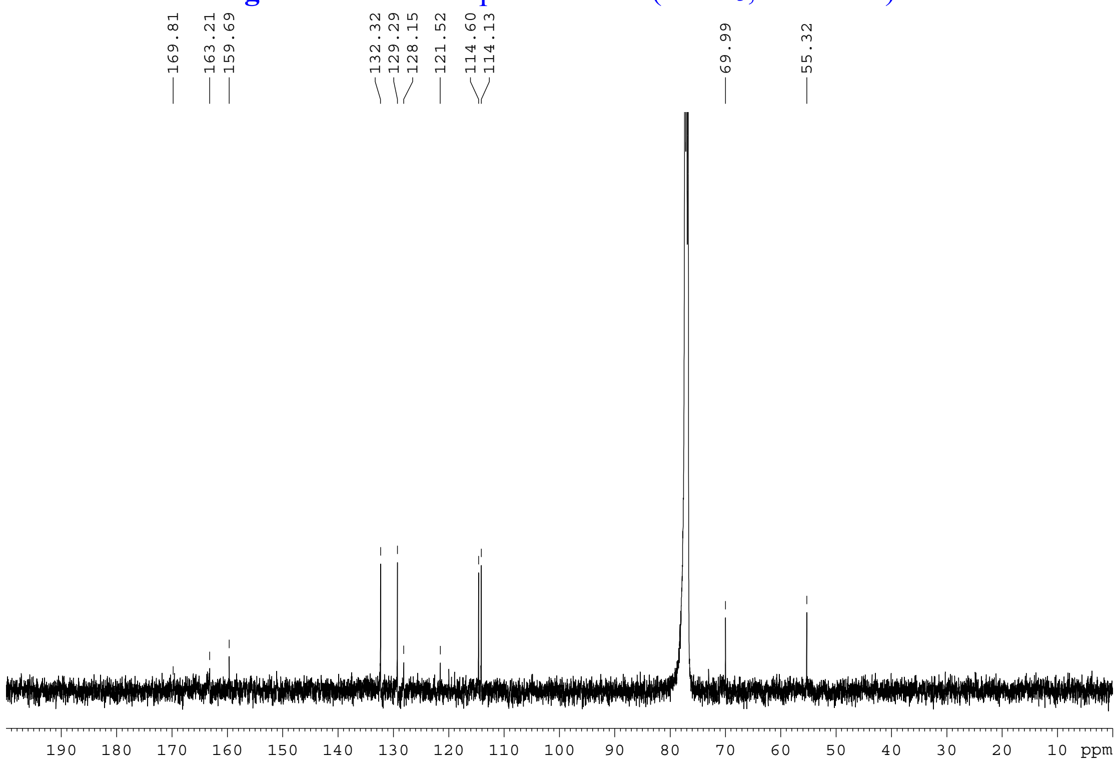

Fig. S17. ^{13}C-NMR spectrum of **3** ($CDCl_3$, 125 MHz).

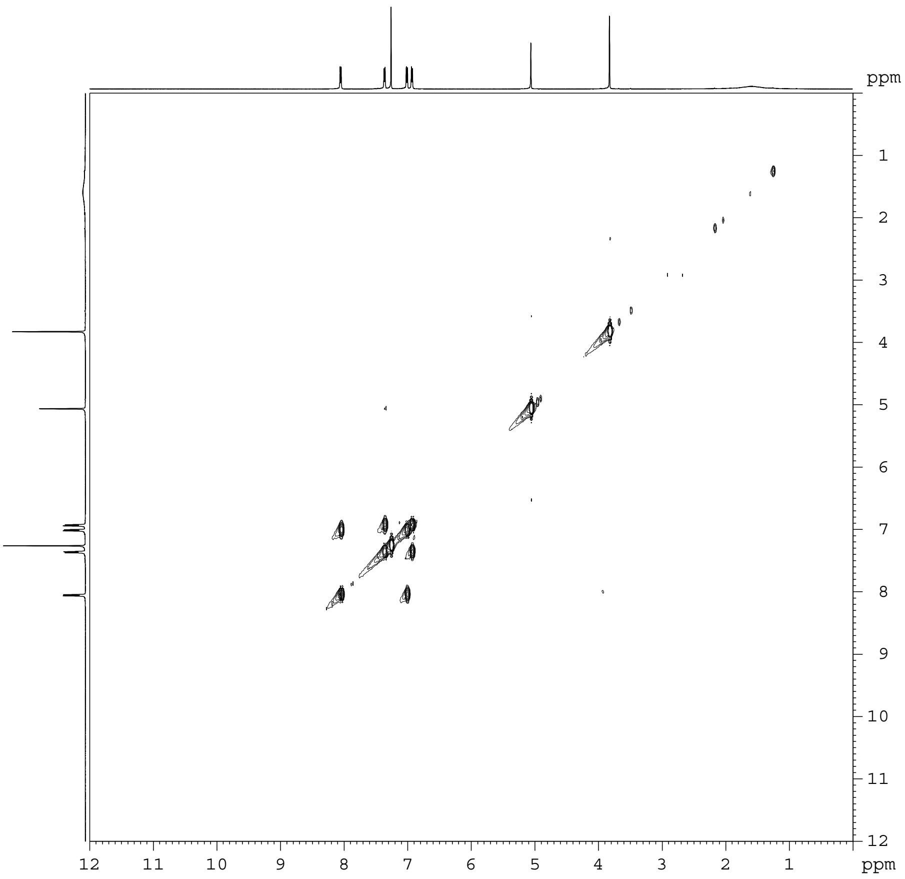

Fig. S18. ^1H–^1H COSY spectrum of **3**.

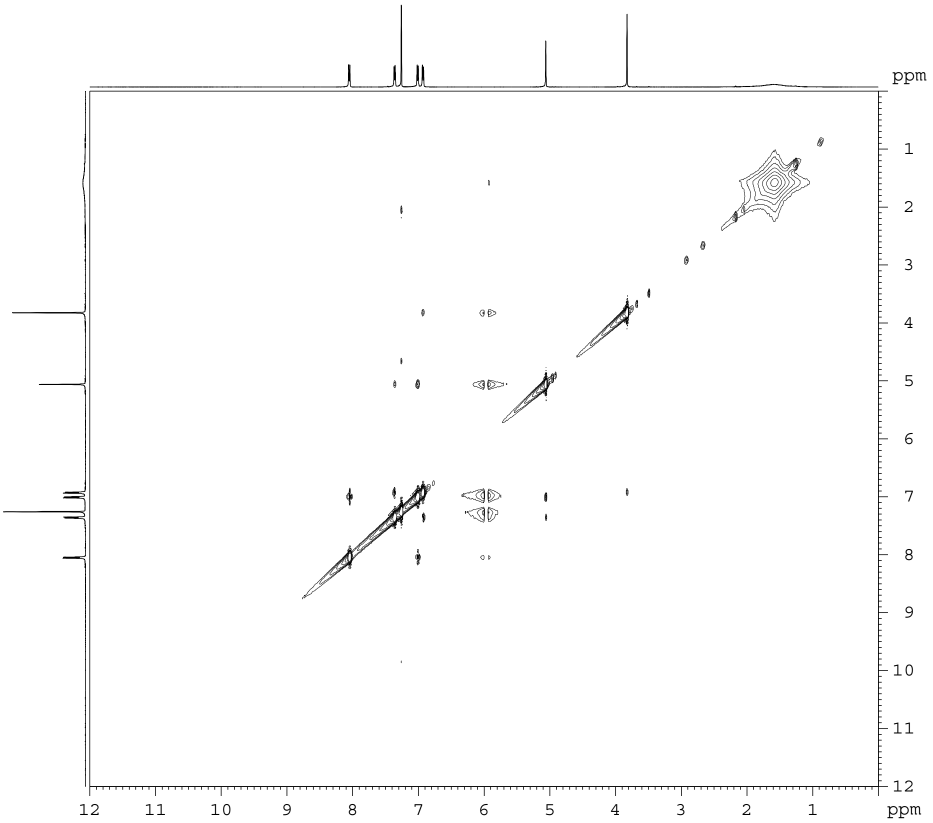

Fig. S19. NOESY spectrum of **3**.

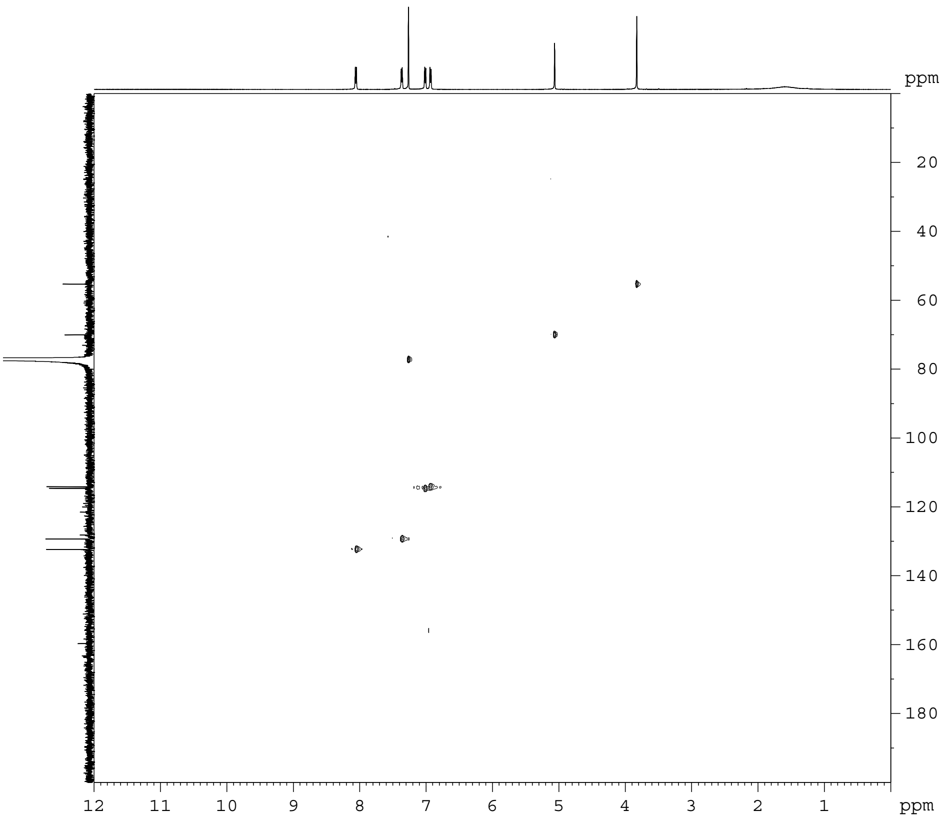

Fig. S20. HSQC spectrum of **3**.

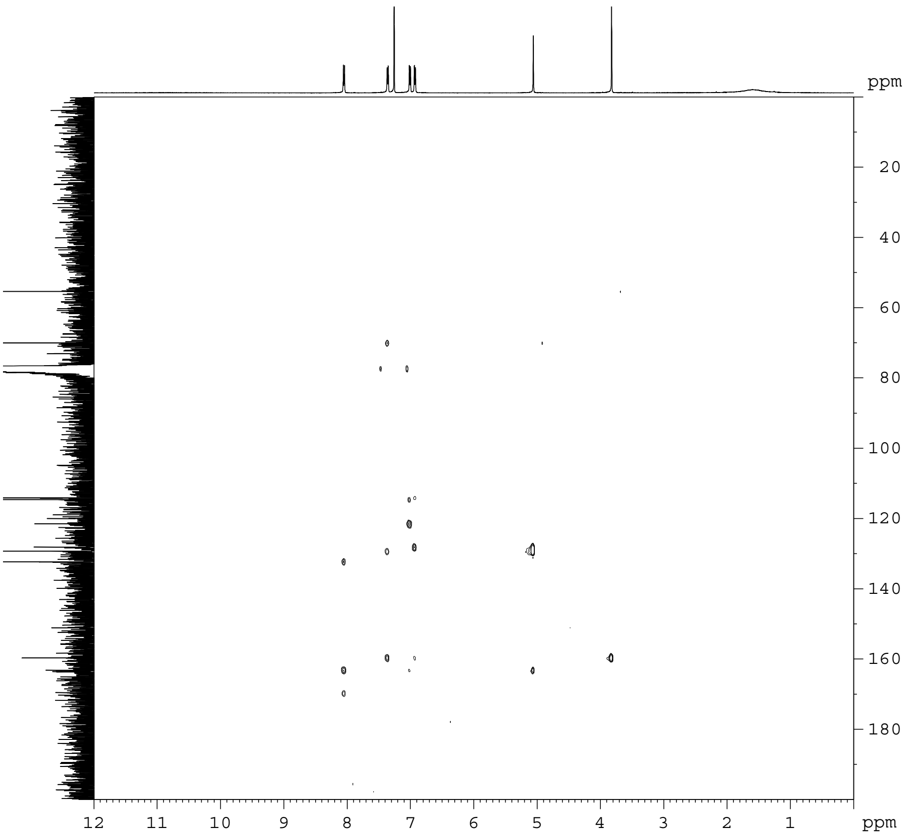

Fig. S21. HMBC spectrum of **3**.
